# Altered Gut Microbiota in a Fragile X Syndrome Mouse Model

**DOI:** 10.3389/fnins.2021.653120

**Published:** 2021-05-26

**Authors:** Francisco Altimiras, José Antonio Garcia, Ismael Palacios-García, Michael J. Hurley, Robert Deacon, Bernardo González, Patricia Cogram

**Affiliations:** ^1^Faculty of Engineering, Pontificia Universidad Católica de Valparaíso, Valparaíso, Chile; ^2^Faculty of Engineering and Business, Universidad de las Américas, Santiago, Chile; ^3^School of Psychology, Pontificia Universidad Católica de Chile, Santiago, Chile; ^4^Centro de Estudios en Neurociencia Humana y Neuropsicología, Facultad de Psicología, Universidad Diego Portales, Santiago, Chile; ^5^Biological Sciences, Faculty of Environmental and Life Sciences, University of Southampton, Southampton, United Kingdom; ^6^Department of Genetics, Institute of Ecology and Biodiversity (IEB), Faculty of Sciences, Universidad de Chile, Santiago, Chile; ^7^FRAXA-DVI, FRAXA Research Foundation, Santiago, Chile; ^8^Faculty of Engineering and Sciences, Universidad Adolfo Ibáñez, Santiago, Chile; ^9^Center of Applied Ecology and Sustainability (CAPES), Santiago, Chile

**Keywords:** autism spectrum disorders, biomarkers, drug development, drug targets, fragile X syndrome, gut microbiota, mouse models, neuroinflammation

## Abstract

The human gut microbiome is the ecosystem of microorganisms that live in the human digestive system. Several studies have related gut microbiome variants to metabolic, immune and nervous system disorders. Fragile X syndrome (FXS) is a neurodevelopmental disorder considered the most common cause of inherited intellectual disability and the leading monogenetic cause of autism. The role of the gut microbiome in FXS remains largely unexplored. Here, we report the results of a gut microbiome analysis using a FXS mouse model and 16S ribosomal RNA gene sequencing. We identified alterations in the fmr1 KO2 gut microbiome associated with different bacterial species, including those in the genera *Akkermansia, Sutterella, Allobaculum, Bifidobacterium, Odoribacter, Turicibacter, Flexispira, Bacteroides*, and *Oscillospira*. Several gut bacterial metabolic pathways were significantly altered in fmr1 KO2 mice, including menaquinone degradation, catechol degradation, vitamin B6 biosynthesis, fatty acid biosynthesis, and nucleotide metabolism. Several of these metabolic pathways, including catechol degradation, nucleotide metabolism and fatty acid biosynthesis, were previously reported to be altered in children and adults with autism. The present study reports a potential association of the gut microbiome with FXS, thereby opening new possibilities for exploring reliable treatments and non-invasive biomarkers.

## Introduction

Fragile X syndrome (FXS) is the leading monogenetic cause of autism spectrum disorder (ASD), a neurodevelopmental condition that affects one in 3,600 males and one in 4,000–6,000 females and currently has no specific drug treatment (Belmonte and Bourgeron, 2006; Hagerman et al., 2009). FXS is a neurodevelopmental condition caused by CGG trinucleotide expansion in the fragile X mental retardation 1 (*Fmr1*) gene locus Xq27 (Levenga et al., 2011; Banerjee et al., 2018). The lack of fragile X mental retardation protein (FMRP) results in a global bias for hypo-editing in the brain of ASD patients leading to dysregulation of synaptic functions and maturation because of altered brain architecture and synaptic pathways (Bagni and Zukin, 2019). Patients with FXS suffer lifelong cognitive deficits, hyperactivity, attention deficit disorder, depression, disturbance in natural behaviors and exhibit autistic traits (Bagni and Zukin, 2019).

A well-characterized model of FXS is the *Fmr1* KO2 mouse, generated by deletion of the promoter and first exon of the Fmr1 gene (Mientjes et al., 2006). *Fmr1* KO2 mice are both protein and mRNA null. *Fmr1* KO2 mice, like the original *Fmr1* KO mice, recapitulate behavioral symptoms observed in humans with FXS, including hyperactivity, repetitive behaviors and deficits in learning and memory (Mientjes et al., 2006). Interestingly both models present anxiety to novel food and reduced flexibility in paradigms that involve task reversal (Kramvis et al., 2013; Kazdoba et al., 2014). *Fmr1* KO2 mice recapitulate the FXS phenotype and represent a preclinical model for the assessment of putative drug treatments (Banerjee et al., 2018). Although our understanding of the physiopathology of FXS has increased in recent years, a disease modifying treatment has not been developed for this condition and current therapies are symptomatic (Pop et al., 2014; Gantois et al., 2019). Thus, more preclinical research is essential for the development of new potential therapeutic agents.

In recent decades, the gut microbiota has emerged as a new research focus for both psychiatric and neurodevelopmental diseases. The trillions of gut microorganisms and their genomes, defined as the human microbiome, contribute to several important processes for human health (Kazdoba et al., 2014). In this context, the human body emerges as a “supra-organism” that presents metabolic traits resulting from the connection between human and microbial genes (Turnbaugh et al., 2007; Peterson et al., 2009). The microbial communities defining the microbiome are principally structural and functional components of the gastrointestinal tract but also of other body parts, such as the mouth, skin and urogenital tract, among others (Turnbaugh et al., 2007; Peterson et al., 2009; Grice and Segre, 2011; Huttenhower et al., 2012). Some of the relevant functions of this community in the gut involve immune response regulation, amino acid metabolism, short-chain fatty acid production, *via* fiber fermentation, and autonomic nervous system regulation *via* the vagus nerve (Cryan and Dinan, 2012). Importantly, a variety of neuroactive compounds, such as serotonin, glutamate, gamma-Aminobutyric acid (GABA) and dopamine, are produced by gut microbiota (Cryan and Dinan, 2012; Cryan et al., 2020). Accordingly, gut microbiome alterations have been implicated in central nervous system (CNS) disorders, including ASD, Alzheimer’s disease, epilepsy, Parkinson’s disease and depression, as well as behavior modulation (Neufeld and Foster, 2009; Rhee et al., 2009; Wikoff et al., 2009; Cryan and O’mahony, 2011; Holmes et al., 2011; Bajaj et al., 2012; Bercik et al., 2012; Collins et al., 2012; Foster and Neufeld, 2013; Mulle et al., 2013; Jiang et al., 2015).

Studies assessing the role of gut microbiota in maintaining normal brain functions have generated encouraging results (Bravo et al., 2011; Bagga et al., 2018; Sgritta et al., 2019) and offer the possibility to develop new therapeutic targets in the treatment of CNS disorders (Grenham et al., 2011; Messaoudi et al., 2011). As a remarkable example of such work, Sampson et al. (2016) demonstrated that alpha-synuclein-dependent motor dysfunction in Parkinson’s disease was reduced after microbial depletion *via* microglial inactivation (Sampson et al., 2016). Moreover, new evidence shows that the therapeutic effects of ketogenic or fasting-mimicking diets on the pathophysiology of Parkinson’s disease are microbiota-dependent (Zhou et al., 2019).

To our knowledge, the relationship between FXS and gut microbiota has not yet been assessed. In this study, we analyzed the gut microbiome composition in a *Fmr1* KO2 mouse model using 16S ribosomal RNA (16S rRNA) gene sequencing. The mouse and human microbiota share 89% similarity in overall bacterial genera meaning the outcome should be realistic and transferable between species when modeling human disease in animal models. We used *Fmr1* KO2 mice to evaluate the composition of the gut microbiota in FXS for later comparison to human FXS patients. Our aim was to explore novel potential tools for translational research. Treatments such as microbiota transfer therapy (MTT) could be a promising tool for ameliorating FXS-related phenotypes. Importantly, the changes identified here in the richness and diversity of the *Fmr1* KO2 microbiota could translate into potential biomarkers for the development and follow-up of potential therapies for FXS patients.

## Materials and Methods

### Animals

In this study, the mice used were *fmr1* KO2 and wild-type (WT) littermates generated on a C57BL/6J background and repeatedly backcrossed onto a C57BL/6J background for more than eight generations. The mice used in this study were provided the FRAXA Research Foundation, MA, United States and by Professor David Nelson from Baylor College.

The *fmr1* KO2 mice were generated by deletion of the promoter and first exon of Fmr1 (Mientjes et al., 2006). The *fmr1* KO2 mice are both, protein and mRNA null. *Fmr1* KO2 mice, like *Fmr1* KO mice, recapitulate behavioral symptoms observed in humans with FXS, including hyperactivity, repetitive behaviors and deficits in learning and memory (Kramvis et al., 2013; Kazdoba et al., 2014).

The mice were housed in 4–5 per cage groups of the same genotype in a temperature- (21 ± 1°C) and humidity-controlled room with a 12-h light–dark cycle (lights on 7 a.m.–7 p.m.). Food and water were available *ad libitum*. Mice were housed in commercial plastic cages on a ventilated rack system.

Experiments were conducted in line with the requirements of the United Kingdom Animals (Scientific Procedures) Act, 1986. All procedures for animal maintenance and experimentation were approved and followed the recommendations of the ethics committee of the Institute of Ecology and Biodiversity (IEB), Faculty of Sciences of the University of Chile, and complied with Chilean regulations.

### Sequencing of the V3–V4 Region of 16S rRNA Gene

Samples for sequencing the V3-V4 region of 16S rRNA gene were obtained from twelve 10 weeks-old male mice of the two genotypes (six FXS and six WT) and from two different sources (six cecum and six feces), with a total of 24 samples sequenced in a Miseq Illumina platform. Samples were collected in 1.5 ml Eppendorf tubes containing 500 μl of nucleic acid stabilization solution (Zymo Research, Irvine, CA, United States), and stored at −80°C until use. DNA was extracted with the DNeasy mini kit (Qiagen, Hilden, Germany) according to the manufacturer’s instructions. DNA concentration and molecular sizes were estimated by UV spectrophotometry and agarose gel electrophoresis. For amplicon preparation, 16S rRNA V3-V4 region was amplified by PCR using 16S amplicon primer pairs: forward = 5′ TCG TCGGCAGCGTCAGATGTGTATAAGAGACAGCCTACGGGN GGCWGCAG 3′, and reverse = 5′ GTCTCGTGGGCTCGGAG ATGTGTATAAGAGACAGGACTACHVGGGTATCTAATCC 3′.

Library preparation was carried out according to the 16S rRNA metagenomics Illumina sequencing library preparation protocol at the Genoma Mayor, Universidad Mayor, Santiago, Chile. The Illumina Miseq platform was used for sequencing of the paired end libraries that were generated. The average sequence depth was 152,983 reads per sample. The average read sequence length was 150 bp before trimming. Average trimmed sequences length was 120 bp. Illumina paired-end data were processed using QIIME (version 1.9.1.) (Caporaso et al., 2010). DADA2 or DEBLUR was used as denoising and normalization strategy. Bacterial taxonomic profiles were obtained using an Operational Taxonomic Units (OTU) picking based analysis (Caporaso et al., 2010; Navas-Molina et al., 2013; Rideout et al., 2014). OTU were clustered against Greengenes 13.8 reference sequences (McDonald et al., 2012) and then subsequently clustered *de novo* at the 97 percent similarity level, using the UClust greedy algorithm.

### Bacterial Diversity Analysis

The average relative abundances of the most highly represented phyla and genera were obtained. We evaluated the global variability of gut bacterial communities in terms of Shannon index, which is a widely used metric for ecological within-sample (alpha) diversity (Buttigieg and Ramette, 2014; Shin et al., 2016). Analogous results were also obtained for other standard metrics for species richness—such as abundance-based coverage estimator (ACE) and Chao1—and alpha diversity (inverse Simpson index). Between-sample (beta) diversity was instead quantified in terms of Bray-Curtis and weighted UniFrac distance, which together capture both the abundance distributions across samples and the phylogenetic relationships. Summarization and visualization of beta diversity were carried out *via* non-metric multi-dimensional scaling (NMDS). All these calculations were performed through the q2-diversity plugin applying the core-metrics-phylogenetic method, computing diversity metrics and generating PCoA plots using Emperor for each of the diversity metrics (Caporaso et al., 2010; Navas-Molina et al., 2013; Rideout et al., 2014). Statistical differences in richness and alpha diversity distributions were estimated with two-tailed Mann–Whitney U tests through the stat_compare_means function in ggplot2. Statistical differences between beta diversity estimates were obtained by permutational multivariate analysis of variance (PERMANOVA) while ensuring the absence of variance inhomogeneity by permutational analysis of multivariate homogeneity of variances (PERMDISP2) through the vegan package in R.

### Microbial Differential Abundance

Differential abundance testing was used to identify OTU that differ between genotypes (WT and FXS). For this purpose, we used the script “group.significance.py” from QIIME (version 1.9.1), to compare OTU frequencies in both sample groups and to ascertain whether or not there were statistically significant differences between the OTU abundance in the different sample groups (Caporaso et al., 2010).

### Metagenome Functional Content Prediction

The PICRUST 2 software package (Langille et al., 2013) was used for predicting functional abundances based on marker gene sequences (16S rRNA sequencing data). MetaCyc ontology predictions (Caspi et al., 2018) were used for metabolic pathways classification.

### Statistics

Non-metric Multidimensional Scaling and Principal Coordinates Analysis (PCoA) were performed in R software environment for statistical computing (R Core Team, 2013). For statistical testing, *t*-test, ANOVA (Analysis of Variance), Mann–Whitney, Kruskal–Wallis, ANOSIM (Analysis of similarities), and PERMANOVA (Permutational multivariate analysis of variance) were used (Caporaso et al., 2010). Bioinformatic analyses including data quality, pre-processing, quantification, and visualization were performed in a Microsoft azure virtual machine with Intel Xeon CPU e5-2673 v3, 2.40 Ghz processor and 32 GB of ram.

## Results

### Bacterial Taxonomic Profiles

**FIGURE 1:**
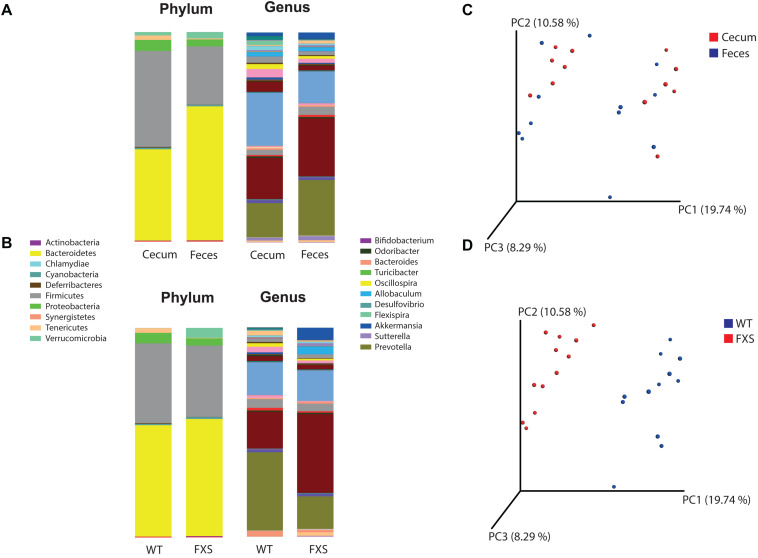
Operational Taxonomic Units (OTU) based analysis according to panel **(A)** sample type and to panel **(B)** genotype, for the phyla and genera taxonomic levels of bacteria. Principal coordinates analysis (PCoA) was used for calculation of beta diversity of the OTU-based bacterial communities. In panel **(C)** are presented the PCoA plots according to the sample type (cecum and feces) and in panel **(D)** to the genotype (FXS and WT).

The bacterial profiles for the sample types (cecum and feces) (Figure 1A) and genotypes (FXS and WT) (Figure 1B) were analyzed. Fifteen bacterial phyla were found in the analyzed samples. The average relative abundances of the most highly represented phyla were Bacteroidetes (54.23%), Firmicutes (36.49%), Proteobacteria (4.49%), Verrucomicrobia (2.33%), Tenericutes (1.27%), and Cyanobacteria (0.73%). Two hundred and two bacterial genera were found in the analyzed samples. The average relative abundances of the most highly represented genera were *Prevotella* (21.3%), *Akkermansia* (2.32%), *Allobaculum* (2.09%), *Oscillospira* (1.76%), *Bacteroides* (1.60%), *Flexispira* (1.1%), *Paraprevotella* (0.81%), *Ruminococcus* (0.78%), *Lactobacillus* (0.76%), *Sutterella* (0.74%), and *Odoribacter* (0.73%).

### Alpha and Beta Diversity Analysis

Alpha diversity was determined for both the sample type and mouse genotype according to the Chao1 rarefaction measure. Significant differences were not observed in the alpha diversity quantification based on a comparison of the mouse genotypes (*p*-value = 0.808, *t*-test statistic = 0.246; *p*-value = 0.131, Mann–Whitney statistic = 98.5) with the sample types (*p*-value = 0.142, *t*-test statistic = 1.524; *p*-value = 0.977, Mann–Whitney statistic = 71).

Beta diversity was analyzed for the *fmr1* KO and WT littermate mice to determine whether some bacterial phyla and genera were differentially abundant based on a comparison of the mouse genotype (WT and FXS) with the sample type (cecum and feces). For the beta diversity analysis, PCoA was used after an OTU picking-based bacterial taxonomy analysis (Figures 1C,D). The comparison of the mouse genotypes showed that samples derived from the FXS group significantly differed from those collected from the WT littermate group (Figure 1D). However, significant differences in the beta diversity metrics were not observed when the sample types were compared, as shown in Figure 1C.

### Bacterial Diversity and Abundance Are Altered in FXS Mice

**FIGURE 2:**
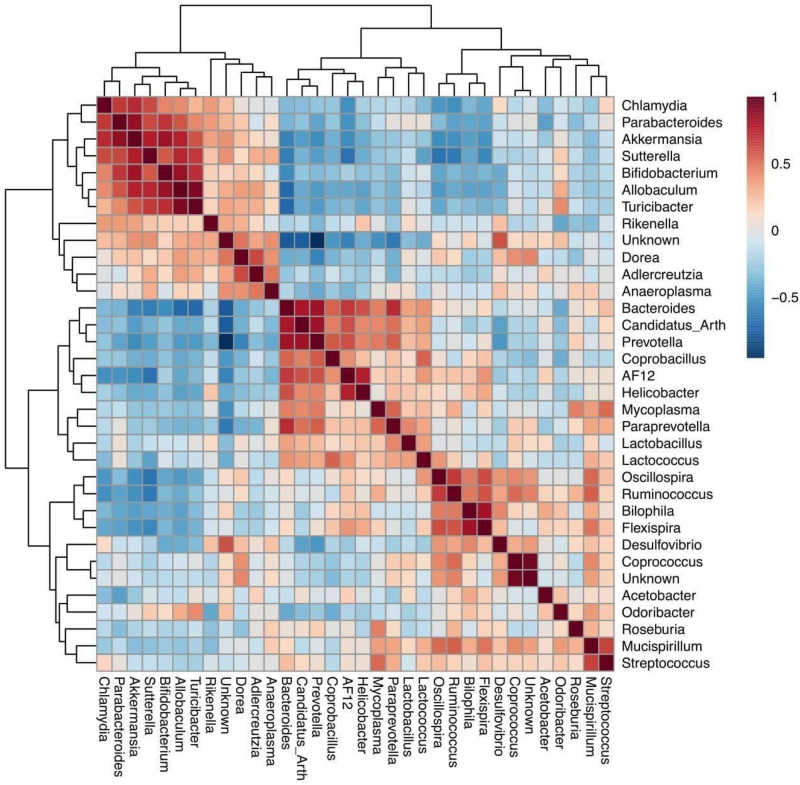
Spearman rank correlation for bacterial genera identified on the *fmr1* KO2 mouse gut microbiome. Red squares indicate a positive correlation, respectively (as is shown in the colored scale).

Differentially abundant bacteria were analyzed between both different mouse genotypes (WT and *Fmr1* KO2). Seven bacterial phyla were found to be significantly differentially abundant: Firmicutes, Tenericutes, Bacteroidetes, Proteobacteria, Verrucomicrobia, Cyanobacteria, and Actinobacteria. Ten bacterial genera were determined to be differentially abundant: *Allobaculum*, *Flexispira*, *Akkermansia*, *Sutterella*, *Bifidobacterium*, *Odoribacter*, *Desulfovibrio*, *Turicibacter*, *Bacteroides*, and *Oscillospira*. Seven of these bacterial genera were increased in *Fmr1* KO2 mice compared with their WT littermates: *Allobaculum*, *Akkermansia*, *Sutterella*, *Bifidobacterium*, *Odoribacter*, *Desulfovibrio*, and *Turicibacter*. Three bacterial genera were decreased: *Flexispira*, *Bacteroides*, and *Oscillospira*. To visualize the correlations among the bacterial genera, a correlation matrix was constructed by calculating all possible pairwise Spearman’s rank correlations (Figure 2).

### Gut Microbiota Metabolites as Potential FXS Biomarkers

**FIGURE 3:**
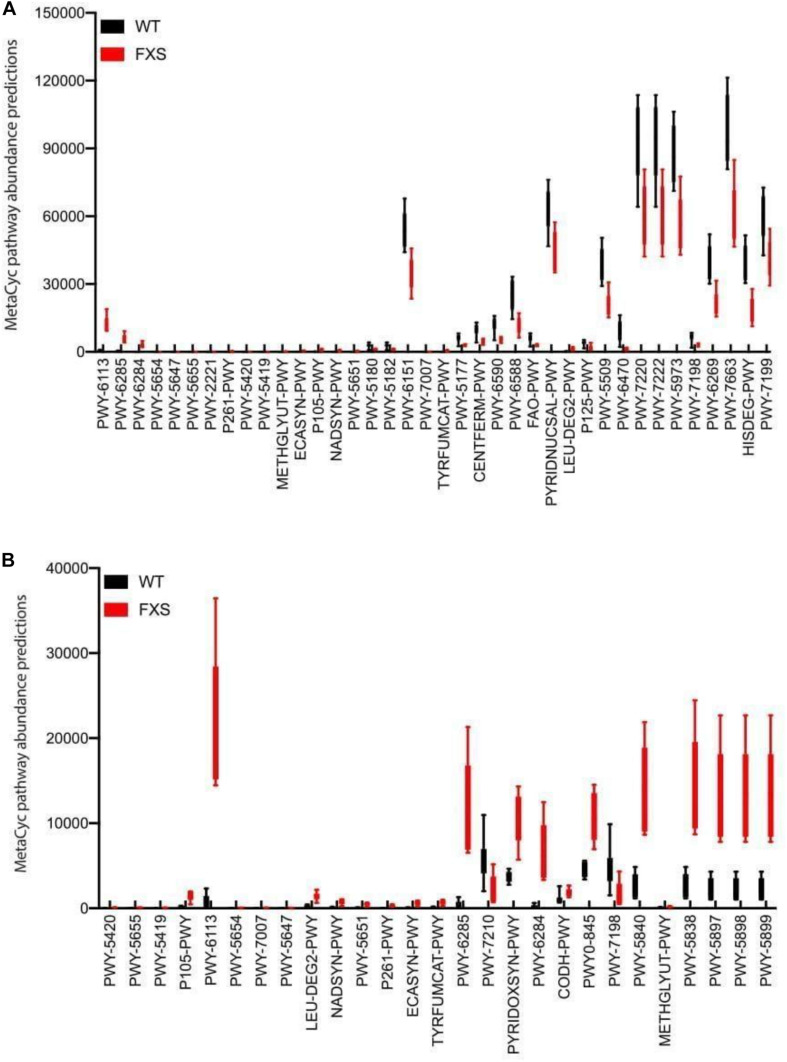
Microbial metabolic pathway prediction on the *fmr1* KO2 mouse gut microbiome. Samples from cecum **(A)** and feces **(B)** were analyzed for the prediction of significantly altered metabolic pathways.

Microbial metabolic pathways were predicted to explore metabolites that might be involved in the dysbiosis observed in the *Fmr1* KO2 mouse gut microbiome. The bioinformatic software package PICRUST2 was used to predict metagenomic functional content from 16S rRNA sequencing data. In Figure 3, MetaCyc pathway abundance predictions are shown for the two types of samples analyzed in this study: cecum (Figure 3A) and feces (Figure 3B). Increased levels of different pathways were observed, including toluene degradation, *S*-adenosyl-*L*-methionine cycle, carboxylate degradation, fermentation to butanoate, fermentation to pyruvate, fatty acid degradation, NAD biosynthesis, pyrimidine nucleotide salvage, and pyrimidine deoxyribonucleotide *de novo* biosynthesis. Decreased levels of different pathways were also observed, including fatty acid biosynthesis, carboxylate degradation, 2-nitrobenzoate degradation, *L*-tryptophan degradation, coenzyme M biosynthesis, catechol degradation, aldehyde degradation, lipopolysaccharide biosynthesis, L-tyrosine degradation, vitamin B6 biosynthesis, and menaquinol biosynthesis.

## Discussion

In the present study, we characterized the *Fmr1* KO2 mouse gut microbiota in terms of taxonomy, diversity and metabolic pathways. To our knowledge, this study presents the first characterization of the *Fmr1* KO2 mouse gut microbiome. The beta diversity analysis showed that the *Fmr1* KO2 microbiome and WT littermate control microbiome showed different bacterial profiles. In addition, we found an increase in the Firmicutes, Bacteroides and Verrucomicrobia phyla, a decrease in the *Prevotella* genus, and an increase in the *Sutterella* and *Akkermansia* genera in the FXS group compared with the WT controls. Increased and decreased metabolic pathways were associated with the FXS microbial profile; thus, the results provide insights on molecular mechanisms that might be involved in the pathology of FXS.

Alterations in the gut microbiome composition have been observed in several neurodevelopmental, psychiatric and gastrointestinal conditions, including depression (Jiang et al., 2015) autism (Sgritta et al., 2019), obesity (Gomes et al., 2018), irritable bowel disease (Liu et al., 2016), and others (Grenham et al., 2011).

In *Fmr1* KO2 mice, the mucin-degrading bacterial genus *Akkermansia*, which belongs to the phylum Verrucomicrobia (Geerlings et al., 2018), increased compared with that in the WT littermate mice, which was consistent with a recently published study that implicated *Akkermansia* in intestinal barrier function (Bedarf et al., 2017).

We identified a significant increase in *Sutterella*, which belongs to the phylum Proteobacteria, in *Fmr1* KO2 mice compared with the WT littermate controls. An increase in *Sutterella* was previously reported in children with ASD (Wang et al., 2013). The abundance of this genus was significantly correlated with the total score of the Aberrant Behavior Checklist (ABC) in Down syndrome persons with ASD (Biagi et al., 2014). The ABC is a widely used measure of ASD behavior (irritability, agitation, lethargy/social withdrawal, stereotypic behavior, hyperactivity and inappropriate speech) and represents an outcome measure for pharmacological studies.

We found that increases in the *Akkermansia* and *Sutterella* genera were positively correlated in a bacterial cluster, suggesting that these bacteria may develop interactive mechanisms for their function in the context of FXS (Banerjee et al., 2018). *Akkermansia* and *Sutterella* interact with the intestinal epithelium and its adhesion properties and the degradation of mucin affects the gut barrier function and proinflammatory capacity (Van Herreweghen et al., 2018). Thus, excessive mucin degradation by these bacteria may facilitate the access of luminal antigens, thereby inducing immunological activation (Hiippala et al., 2016). The immunomodulatory role of these bacteria on mucosal barrier function might be a possible bacterial-host interaction mechanism that promotes disease progression in FXS patients (Ganesh et al., 2013; Azhari et al., 2019).

Interestingly, a recent study showed that *Akkermansia* plays a role (directly or indirectly) in hippocampal function by regulating proinflammatory cytokine expression, neuronal development and plasticity and hippocampus-related cognitive processes (Derrien et al., 2017; Yang et al., 2019). Similarly, the *Fmr1* KO2 mouse model was characterized by disruption of hippocampal neurogenesis, synaptic plasticity and neuronal communication, thus reflecting a possible mechanism underlying the cognitive impairments in FXS and similar autistic conditions (Bostrom et al., 2016). *Akkermansia* may therefore play a role in the regulation of hippocampal function and cognitive processes associated with FXS. However, more work assessing hippocampal function along with the microbiota in the FXS context is needed to confirm this point. Moreover, the putative role of *Akkermansia* in the maintenance of the gut barrier and host metabolism must be highlighted (Derrien et al., 2017). Our results are consistent with those of Ganesh et al. (2013), who showed that increased levels of *Akkermansia* induce gut barrier disruption and activate an inflammatory response associated with the psychopathology of conditions such as autism (Ganesh et al., 2013; Van Herreweghen et al., 2018; Azhari et al., 2019). Similarly, postmortem exams of autistic patients have identified gut barrier disruption (Fiorentino et al., 2016) suggesting that autism may be influenced by gut integrity throughout life and modification of the gut microbiome might be of benefit in patients with the disorder (Azhari et al., 2019). Similar to our results, previous studies shown increased levels of *Akkermansia* species in animal models and patients with autism. In Newell et al. (2016) the authors identified elevated *Akkermansia muciniphila* content in the cecal and fecal matter of BTBRT + tf/j (BTBR) mouse model of ASD. Moreover, in De Angelis et al. (2013), *Akkermansia* species were almost the highest in autistic children. Therefore, we hypothesize that *A. muciniphila*, possessing the ability to disturb host mucus-homeostasis, appears to play a role in FXS through increased intestinal permeability and inflammation. However, more studies are needed to validate whether the abundance of species of the genus *Akkermansia* are relevant for patients with FXS.

A predictive functional metagenomics analysis was carried out using a bioinformatic approach to characterize the metabolic pathways that might be affected in the *Fmr1* KO2 model, thereby shedding light on the possible molecular mechanisms underlying the effects of the microbiota on the psychopathology of FXS. Several metabolic pathways, such as the pyrimidine, adenosine and guanosine biosynthesis pathways, were decreased in this model, which is consistent with other results using animal models of autism (Page, 2000; Page and Coleman, 2000) and metabolomic analyses of these models (Naviaux et al., 2014). In addition, we found that fatty acid biosynthesis was increased in the FXS group, in both the cecum and fecal samples. Altered gut microbiota and increased short-chain fatty acids were previously reported in children with ASD (Meguid et al., 2008; MacFabe, 2012; Wang et al., 2012; Zhang et al., 2018). Moreover, increased levels of *Akkermansia* were also shown to be related to the augmentation of short-chain fatty acids, suggesting that the regulation of fatty acid metabolism by *Akkermansia* might be involved in FXS (Puertollano et al., 2014; Ottman et al., 2017). Furthermore, predicted pathways related to menaquinol (vitamin K2) biosynthesis were increased in the FXS group. Menaquinone has been shown to be essential in electron transport and ATP generation in all gram-positive and anaerobically respiring gram-negative bacteria. Additionally, menaquinone is also essential in maintaining normal neural development (Adams et al., 2011). Specific neural effects of vitamin K overlap with key brain development aberrations, including those associated with autism (Adams et al., 2011). This evidence might be associated with the hippocampal disruption observed in FXS animal models (Arbab et al., 2018).

The observed abundance of *Allobaculum* in *Fmr1* KO2 mice could produce a negative balance of circulating leptin. *Allobaculum* species presence is negatively correlated with the level of leptin, which has an important role in the immune system controlling glucose homeostasis, the autonomic nervous system and the neuroendocrine axes. Consistent with our results on *Fmr1* KO2 mice, previous reports found that ASD children show a significantly higher prevalence of *Sutterella* species compared to the control group (Williams et al., 2012). Interestingly, the genera *Bacteroides* and *Oscillospira* were suggested to be associated with the pathological traits observed in the BTBR mouse model of autism (Coretti et al., 2017).

The present findings are novel and informative in the process of understanding the potential role of the microbiome in FXS. First, additional human studies are needed to fully characterize the translational power and potential role of the microbiome in FXS. Second, work exploring the correlation between microbiota and behavior in animal models and FXS patients would be useful to better understand the clinical implications and generate less speculative conclusions. Finally, given that FXS syndrome is seemingly influenced by brain-gut microbiota pathways, more exhaustive experiments that include brain gene expression, electroencephalography measurements and the use of compounds known to restore the normal FXS phenotype, such as metformin, may contribute to untangling the complexity of FXS. The use of probiotic candidates could become a powerful tool to help ameliorate the FXS phenotype. Our results provide new insights into the interactions between the brain and the gut microbiome in FXS. It seems that certain bacterial genera of the gut microbiome could interact at the level of the gastrointestinal barrier. The evidence presented above, in addition to the gastrointestinal disturbance observed in patients with autism, suggests a novel and interesting line of research in FXS. Further studies using larger sample sizes and microbial detection at the species level are required to elucidate the potential contribution of the microbiota as a biomarker and treatment target in FXS.

## Data Availability Statement

Raw sequencing data is available through the NCBI, using accession PRJNA611542.

## Ethics Statement

The animal study was reviewed and approved by Institute of Ecology and Biodiversity (IEB), Ethical Committee, headed by Professor Rodrigo Vasquez. Written informed consent was obtained from the owners for the participation of their animals in this study.

## Author Contributions

FA, PC, and BG contributed with the conception, design of the study, data analysis, interpretation, and drafting the manuscript. JG and IP-G contributed with the data analysis, interpretation, and drafting of the manuscript. MH and RD contributed with the data analysis, interpretation and critical revision of the manuscript. All authors contributed to the article and approved the submitted version.

## Conflict of Interest

The authors declare that the research was conducted in the absence of any commercial or financial relationships that could be construed as a potential conflict of interest.

## References

[B1] Adams J. B. Audhya T. McDonough-Means S. Rubin R. A. Quig D. Geis E. (2011). Effect of a vitamin/mineral supplement on children and adults with autism. *BMC Pediatr.* 11:111. 10.1186/1471-2431-11-111 22151477 PMC3266205

[B2] Arbab T. Pennartz C. M. Battaglia F. P. (2018). Impaired hippocampal representation of place in the Fmr1-knockout mouse model of fragile X syndrome. *Sci. Rep.* 8:8889. 10.1038/s41598-018-26853-z PMC5995880 29892074

[B3] Azhari A. Azizan F. Esposito G. (2019). A systematic review of gut-immune-brain mechanisms in Autism Spectrum Disorder. *Dev. Psychobiol.* 61 752–771. 10.1002/dev.21803 30523646

[B4] Bagga D. Reichert J. L. Koschutnig K. Aigner C. S. Holzer P. Koskinen K. (2018). Probiotics drive gut microbiome triggering emotional brain signatures. *Gut Microbes* 9 486–496. 29723105 10.1080/19490976.2018.1460015 PMC6287679

[B5] Bagni C. Zukin R. S. (2019). A synaptic perspective of fragile X syndrome and autism spectrum disorders. *Neuron* 101 1070–1088. 10.1016/j.neuron.2019.02.041 30897358 PMC9628679

[B6] Bajaj J. S. Ridlon J. M. Hylemon P. B. Thacker L. R. Heuman D. M. Smith S. (2012). Linkage of gut microbiome with cognition in hepatic encephalopathy. *Am. J. Physiol.-Gastrointestinal Liver Physiol.* 302 G168–G175. 10.1152/ajpgi.00190.2011 PMC3345956 21940902

[B7] Banerjee A. Ifrim M. F. Valdez A. N. Raj N. Bassell G. J. (2018). Aberrant RNA translation in fragile X syndrome: from FMRP mechanisms to emerging therapeutic strategies. *Brain Res.* 1693 24–36. 10.1016/j.brainres.2018.04.008 29653083 PMC7377270

[B8] Bedarf J. R. Hildebrand F. Coelho L. P. Sunagawa S. Bahram M. Goeser F. (2017). Functional implications of microbial and viral gut metagenome changes in early stage L-DOPA-naïve Parkinson’s disease patients. *Genome Med.* 9:39. 10.1186/s13073-017-0428-y PMC5408370 28449715

[B9] Belmonte M. K. Bourgeron T. (2006). Fragile X syndrome and autism at the intersection of genetic and neural networks. *Nat. Neurosci.* 9 1221–1225. 10.1038/nn1765 17001341

[B10] Bercik P. Collins S. M. Verdu E. F. (2012). Microbes and the gut-brain axis. *Neurogastroenterol. Motil.* 24 405–413. 10.1111/j.1365-2982.2012.01906.x 22404222

[B11] Biagi E. Candela M. Centanni M. Consolandi C. Rampelli S. Turroni S. (2014). Gut microbiome in down syndrome. *PLoS One* 9:e112023. 10.1371/journal.pone.0112023 25386941 PMC4227691

[B12] Bostrom C. Yau S. Y. Majaess N. Vetrici M. Gil-Mohapel J. Christie B. R. (2016). Hippocampal dysfunction and cognitive impairment in Fragile-X Syndrome. *Neurosci. Biobehav. Rev.* 68 563–574. 10.1016/j.neubiorev.2016.06.033 27345143

[B13] Bravo J. A. Forsythe P. Chew M. V. Escaravage E. Savignac H. M. Dinan T. G. (2011). Ingestion of *Lactobacillus* strain regulates emotional behavior and central GABA receptor expression in a mouse via the vagus nerve. *Proc. Natl. Acad. Sci. U S A.* 108 16050–16055. 10.1073/pnas.1102999108 21876150 PMC3179073

[B14] Buttigieg P. L. Ramette A. (2014). A guide to statistical analysis in microbial ecology: a community-focused, living review of multivariate data analyses. *FEMS Microbiol. Ecol.* 90 543–550. 10.1111/1574-6941.12437 25314312

[B15] Caporaso J. G. Kuczynski J. Stombaugh J. Bittinger K. Bushman F. D. Costello E. K. (2010). QIIME allows analysis of high-throughput community sequencing data. *Nat. Methods* 7 335–336. 20383131 10.1038/nmeth.f.303 PMC3156573

[B16] Caspi R. Billington R. Fulcher C. A. Keseler I. M. Kothari A. Krummenacker M. (2018). The MetaCyc database of metabolic pathways and enzymes. *Nucleic Acids Res.* 46 D633–D639. 29059334 10.1093/nar/gkx935 PMC5753197

[B17] Collins S. M. Surette M. Bercik P. (2012). The interplay between the intestinal microbiota and the brain. *Nat. Rev. Microbiol.* 10 735–742. 10.1038/nrmicro2876 23000955

[B18] Coretti L. Cristiano C. Florio E. Scala G. Lama A. Keller S. (2017). Sex-related alterations of gut microbiota composition in the BTBR mouse model of autism spectrum disorder. *Sci. Rep.* 7:45356. 10.1038/srep45356 PMC5368984 28349974

[B19] Cryan J. F. Dinan T. G. (2012). Mind-altering microorganisms: the impact of the gut microbiota on brain and behaviour. *Nat. Rev. Neurosci.* 13 701–712. 10.1038/nrn3346 22968153

[B20] Cryan J. F. O’mahony S. M. (2011). The microbiome-gut-brain axis: from bowel to behavior. *Neurogastroenterol. Motil.* 23 187–192. 10.1111/j.1365-2982.2010.01664.x 21303428

[B21] Cryan J. F. O’Riordan K. J. Sandhu K. Peterson V. Dinan T. G. (2020). The gut microbiome in neurological disorders. *Lancet Neurol.* 19 179–194. 31753762 10.1016/S1474-4422(19)30356-4

[B22] De Angelis M. Piccolo M. Vannini L. Siragusa S. De Giacomo A. Serrazzanetti D. I. (2013). Fecal microbiota and metabolome of children with autism and pervasive developmental disorder not otherwise specified. *PLoS One* 8:e76993. 10.1371/journal.pone.0076993 24130822 PMC3793965

[B23] Derrien M. Belzer C. de Vos W. M. (2017). Akkermansia muciniphila and its role in regulating host functions. *Microb. Pathog.* 106 171–181. 10.1016/j.micpath.2016.02.005 26875998

[B24] Fiorentino M. Sapone A. Senger S. Camhi S. S. Kadzielski S. M. Buie T. M. (2016). Blood–brain barrier and intestinal epithelial barrier alterations in autism spectrum disorders. *Mol. Autism* 7:49. 10.1186/s13229-016-0110-z PMC5129651 27957319

[B25] Foster J. A. Neufeld K. A. M. (2013). Gut–brain axis: how the microbiome influences anxiety and depression. *Trends Neurosci.* 36 305–312. 10.1016/j.tins.2013.01.005 23384445

[B26] Ganesh B. P. Klopfleisch R. Loh G. Blaut M. (2013). Commensal Akkermansia muciniphila exacerbates gut inflammation in *Salmonella* Typhimurium-infected gnotobiotic mice. *PLoS One* 8:e74963. 10.1371/journal.pone.0074963 24040367 PMC3769299

[B27] Gantois I. Popic J. Khoutorsky A. Sonenberg N. (2019). Metformin for treatment of fragile X syndrome and other neurological disorders. *Annu. Rev. Med.* 70 167–181. 10.1146/annurev-med-081117-041238 30365357

[B28] Geerlings S. Y. Kostopoulos I. De Vos W. M. Belzer C. (2018). Akkermansia muciniphila in the human gastrointestinal tract: when, where, and how? *Microorganisms* 6:75. 10.3390/microorganisms6030075 30041463 PMC6163243

[B29] Gomes A. C. Hoffmann C. Mota J. F. (2018). The human gut microbiota: metabolism and perspective in obesity. *Gut Microbes* 9 308–325. 29667480 10.1080/19490976.2018.1465157 PMC6219651

[B30] Grenham S. Clarke G. Cryan J. F. Dinan T. G. (2011). Brain–gut–microbe communication in health and disease. *Front. Physiol.* 2:94. 10.3389/fphys.2011.00094 22162969 PMC3232439

[B31] Grice E. A. Segre J. A. (2011). The skin microbiome. *Nat. Rev. Microbiol.* 9 244–253. 21407241 10.1038/nrmicro2537 PMC3535073

[B32] Hagerman R. J. Berry-Kravis E. Kaufmann W. E. Ono M. Y. Tartaglia N. Lachiewicz A. (2009). Advances in the treatment of fragile X syndrome. *Pediatrics* 123 378–390. 19117905 10.1542/peds.2008-0317 PMC2888470

[B33] Hiippala K. Kainulainen V. Kalliomäki M. Arkkila P. Satokari R. (2016). Mucosal prevalence and interactions with the epithelium indicate commensalism of Sutterella s. *Front. Microbiol.* 7:1706. 10.3389/fmicb.2016.01706 27833600 PMC5080374

[B34] Holmes E. Li J. V. Athanasiou T. Ashrafian H. Nicholson J. K. (2011). Understanding the role of gut microbiome–host metabolic signal disruption in health and disease. *Trends Microbiol.* 19 349–359. 10.1016/j.tim.2011.05.006 21684749

[B35] Huttenhower C. Gevers D. Knight R. Abubucker S. Badger J. H. Chinwalla A. T. (2012). Structure, function and diversity of the healthy human microbiome. *Nature* 486:207. 10.1038/nature11234 22699609 PMC3564958

[B36] Jiang H. Ling Z. Zhang Y. Mao H. Ma Z. Yin Y. (2015). Altered fecal microbiota composition in patients with major depressive disorder. *Brain Behav. Immun.* 48 186–194. 10.1016/j.bbi.2015.03.016 25882912

[B37] Kazdoba T. M. Leach P. T. Silverman J. L. Crawley J. N. (2014). Modeling fragile X syndrome in the Fmr1 knockout mouse. *Intractable Rare Dis. Res.* 3 118–133. 10.5582/irdr.2014.01024 25606362 PMC4298642

[B38] Kramvis I. Mansvelder H. Loos M. Meredith R. (2013). Hyperactivity, perseveration and increased responding during attentional rule acquisition in the Fragile X mouse model. *Front. Behav. Neurosci.* 7:172. 10.3389/fnbeh.2013.00172 24312033 PMC3836024

[B39] Langille M. G. Zaneveld J. Caporaso J. G. McDonald D. Knights D. Reyes J. A. (2013). Predictive functional profiling of microbial communities using 16S rRNA marker gene sequences. *Nat. Biotechnol.* 31 814–821. 10.1038/nbt.2676 23975157 PMC3819121

[B40] Levenga J. Hayashi S. de Vrij F. M. Koekkoek S. K. van der Linde H. C. Nieuwenhuizen I. (2011). AFQ056, a new mGluR5 antagonist for treatment of fragile X syndrome. *Neurobiol. Dis.* 42 311–317. 10.1016/j.nbd.2011.01.022 21316452

[B41] Liu Y. Zhang L. (2016). Similar fecal microbiota signatures in patients with diarrhea-predominant irritable bowel syndrome and patients with depression. *Clin. Gastroenterol. Hepatol.* 14 1602–1611. 10.1016/j.cgh.2016.05.033 27266978

[B42] MacFabe D. F. (2012). Short-chain fatty acid fermentation products of the gut microbiome: implications in autism spectrum disorders. *Microbial Ecol. Health Dis.* 23:19260. 10.3402/mehd.v23i0.19260 PMC3747729 23990817

[B43] McDonald D. Price M. N. Goodrich J. Nawrocki E. P. DeSantis T. Z. Probst A. (2012). An improved Greengenes taxonomy with explicit ranks for ecological and evolutionary analyses of bacteria and archaea. *ISME J.* 6 610–618. 10.1038/ismej.2011.139 22134646 PMC3280142

[B44] Meguid N. A. Atta H. M. Gouda A. S. Khalil R. O. (2008). Role of polyunsaturated fatty acids in the management of Egyptian children with autism. *Clin. Biochem.* 41 1044–1048. 10.1016/j.clinbiochem.2008.05.013 18582451

[B45] Messaoudi M. Violle N. Bisson J. F. Desor D. Javelot H. Rougeot C. (2011). Beneficial psychological effects of a probiotic formulation (Lactobacillus helveticus R0052 and Bifidobacterium longum R0175) in healthy human volunteers. *Gut Microbes* 2 256–261. 10.4161/gmic.2.4.16108 21983070

[B46] Mientjes E. J. Nieuwenhuizen I. Kirkpatrick L. Zu T. Hoogeveen-Westerveld M. Severijnen L. (2006). The generation of a conditional Fmr1 knock out mouse model to study Fmrp function in vivo. *Neurobiol. Dis.* 21 549–555. 10.1016/j.nbd.2005.08.019 16257225

[B47] Mulle J. G. Sharp W. G. Cubells J. F. (2013). The gut microbiome: a new frontier in autism research. *Curr. Psychiatry Rep.* 15:337. 10.1007/s11920-012-0337-0 PMC3564498 23307560

[B48] Navas-Molina J. A. Peralta-Sánchez J. M. González A. McMurdie P. J. Vázquez-Baeza Y. Xu Z. (2013). Advancing our understanding of the human microbiome using QIIME. *Methods Enzymol.* 531 371–444. 10.1016/b978-0-12-407863-5.00019-8 24060131 PMC4517945

[B49] Naviaux J. C. Schuchbauer M. A. Li K. Wang L. Risbrough V. B. Powell S. B. (2014). Reversal of autism-like behaviors and metabolism in adult mice with single-dose antipurinergic therapy. *Transl. Psychiatry* 4:e400. 10.1038/tp.2014.33 24937094 PMC4080315

[B50] Neufeld K. A. Foster J. A. (2009). Effects of gut microbiota on the brain: implications for psychiatry. *J. Psychiatry Neurosci.: JPN* 34:230. PMC2674977 19448854

[B51] Newell C. Bomhof M. R. Reimer R. A. Hittel D. S. Rho J. M. Shearer J. (2016). Ketogenic diet modifies the gut microbiota in a murine model of autism spectrum disorder. *Mol. Autism* 7:37. 10.1186/s13229-016-0099-3 PMC5009541 27594980

[B52] Ottman N. Geerlings S. Y. Aalvink S. de Vos W. M. Belzer C. (2017). Action and function of Akkermansia muciniphila in microbiome ecology, health and disease. *Best Pract. Res. Clin. Gastroenterol.* 31 637–642. 10.1016/j.bpg.2017.10.001 29566906

[B53] Page T. (2000). Metabolic approaches to the treatment of autism spectrum disorders. *J. Autism. Dev. Disord.* 30 463–469. 11098885 10.1023/a:1005563926383

[B54] Page T. Coleman M. (2000). Purine metabolism abnormalities in a hyperuricosuric subclass of autism. *Biochim. et Biophys. Acta (BBA)-Mol. Basis Dis.* 1500 291–296. 10.1016/s0925-4439(99)00113-1 10699370

[B55] Peterson J. Garges S. Giovanni M. McInnes P. Wang L. Schloss J. A. (2009). The NIH human microbiome project. *Genome Res.* 19 2317–2323. 19819907 10.1101/gr.096651.109 PMC2792171

[B56] Pop A. S. Gomez-Mancilla B. Neri G. Willemsen R. Gasparini F. (2014). Fragile X syndrome: a preclinical review on metabotropic glutamate receptor 5 (mGluR5) antagonists and drug development. *Psychopharmacology* 231 1217–1226. 10.1007/s00213-013-3330-3 24232444

[B57] Puertollano E. Kolida S. Yaqoob P. (2014). Biological significance of short-chain fatty acid metabolism by the intestinal microbiome. *Curr. Opin. Clin. Nutrit. Metab. Care* 17 139–144. 10.1097/mco.0000000000000025 24389673

[B58] R Core Team (2013). *R: A Language and Environment for Statistical Computing.* Vienna: R Core Team.

[B59] Rhee S. H. Pothoulakis C. Mayer E. A. (2009). Principles and clinical implications of the brain–gut–enteric microbiota axis. *Nat. Rev. Gastroenterol. Hepatol.* 6:306. 10.1038/nrgastro.2009.35 19404271 PMC3817714

[B60] Rideout J. R. He Y. Navas-Molina J. A. Walters W. A. Ursell L. K. Gibbons S. M. (2014). Subsampled open-reference clustering creates consistent, comprehensive OTU definitions and scales to billions of sequences. *PeerJ* 2:e545. 10.7717/peerj.545 25177538 PMC4145071

[B61] Sampson T. R. Debelius J. W. Thron T. Janssen S. Shastri G. G. Ilhan Z. E. (2016). Gut microbiota regulate motor deficits and neuroinflammation in a model of Parkinson’s disease. *Cell* 167 1469–1480. 27912057 10.1016/j.cell.2016.11.018 PMC5718049

[B62] Sgritta M. Dooling S. W. Buffington S. A. Momin E. N. Francis M. B. Britton R. A. (2019). Mechanisms underlying microbial-mediated changes in social behavior in mouse models of autism spectrum disorder. *Neuron* 101 246–259. 10.1016/j.neuron.2018.11.018 30522820 PMC6645363

[B63] Shin J. Lee S. Go M. J. Lee S. Y. Kim S. C. Lee C. H. (2016). Analysis of the mouse gut microbiome using full-length 16S rRNA amplicon sequencing. *Sci. Rep.* 6:29681. 10.1038/srep29681 PMC4944186 27411898

[B64] Turnbaugh P. J. Ley R. E. Hamady M. Fraser-Liggett C. M. Knight R. Gordon J. I. (2007). The human microbiome project. *Nature* 449 804–810. 17943116 10.1038/nature06244 PMC3709439

[B65] Van Herreweghen F. De Paepe K. Roume H. Kerckhof F. M. Van de Wiele T. (2018). Mucin degradation niche as a driver of microbiome composition and Akkermansia muciniphila abundance in a dynamic gut model is donor independent. *FEMS Microbiol. Ecol.* 94:fiy186. 10.1093/femsec/fiy186 30239657

[B66] Wang L. Christophersen C. T. Sorich M. J. Gerber J. P. Angley M. T. Conlon M. A. (2012). Elevated fecal short chain fatty acid and ammonia concentrations in children with autism spectrum disorder. *Dig. Dis. Sci.* 57 2096–2102. 10.1007/s10620-012-2167-7 22535281

[B67] Wang L. Christophersen C. T. Sorich M. J. Gerber J. P. Angley M. T. Conlon M. A. (2013). Increased abundance of Sutterella s and Ruminococcus torques in feces of children with autism spectrum disorder. *Mol. Autism* 4:42. 10.1186/2040-2392-4-42 24188502 PMC3828002

[B68] Wikoff W. R. Anfora A. T. Liu J. Schultz P. G. Lesley S. A. Peters E. C. (2009). Metabolomics analysis reveals large effects of gut microflora on mammalian blood metabolites. *Proc. Natl. Acad. Sci. U S A.* 106 3698–3703. 10.1073/pnas.0812874106 19234110 PMC2656143

[B69] Williams B. L. Hornig M. Parekh T. Lipkin W. I. (2012). Application of novel PCR-based methods for detection, quantitation, and phylogenetic characterization of Sutterella species in intestinal biopsy samples from children with autism and gastrointestinal disturbances. *mBio* 3:e00261-11. 10.1128/mBio.00261-11 PMC3252763 22233678

[B70] Yang Y. Zhong Z. Wang B. Xia X. Yao W. Huang L. (2019). Early-life high-fat diet-induced obesity programs hippocampal development and cognitive functions via regulation of gut commensal Akkermansia muciniphila. *Neuropsychopharmacology* 44 2054–2064. 10.1038/s41386-019-0437-1 31207607 PMC6897910

[B71] Zhang M. Ma W. Zhang J. He Y. Wang J. (2018). Analysis of gut microbiota profiles and microbe-disease associations in children with autism spectrum disorders in China. *Sci. Rep.* 8:13981. 10.1038/s41598-018-32219-2 PMC6143520 30228282

[B72] Zhou Z. L. Jia X. B. Sun M. F. Zhu Y. L. Qiao C. M. Zhang B. P. (2019). Neuroprotection of fasting mimicking diet on MPTP-induced Parkinson’s disease mice via gut microbiota and metabolites. *Neurotherapeutics* 16 741–760. 10.1007/s13311-019-00719-2 30815845 PMC6694382

